# Hepatic Epithelioid Hemangioendothelioma Mimicking Liver Metastases in a Young Woman

**DOI:** 10.5334/jbsr.3578

**Published:** 2024-05-03

**Authors:** Jarno De Craemer, Bart Mortelé, Bart Lutin

**Affiliations:** 1 AZ Groeninge, Kortrijk, Belgium; 2 AZ Groeninge, Kortrijk, Belgium; 3 AZ Groeninge, Kortrijk, Belgium

**Keywords:** epithelioid hemangioendothelioma, multifocal liver lesions

## Abstract

*Teaching point:* When confronted with multifocal “metastasis-like” liver lesions without a known primary tumor, in particular in younger female patients, considering hepatic epithelioid hemangioendothelioma (HEHE) in the differential diagnosis can guide pathological examination and potentially avoid the need for multiple invasive biopsies.

## Case History

**Figure 1:**
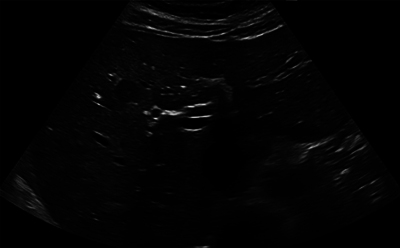
Ultrasound of the liver showing hypoechogenic nodular lesions.

**Figure 2:**
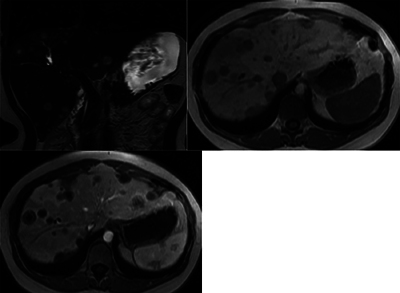
MRI showing multiple, coalescent liver lesions with target appearance, peripheral enhancement and central necrosis (upper left: T2WI; upper right: T1WI; lower left: T1WI + Gd).

**Figure 3:**
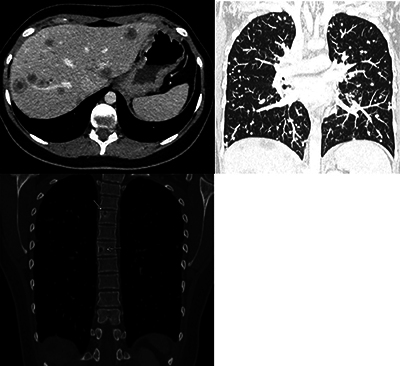
CT showing multiple, coalescent liver lesions with target appearance and peripheral enhancement (upper left), diffusely spread lung nodules (upper right) and multiple osteolytic bone lesions (lower left).

A 21-year-old woman presented with a 1-month history of mild fatigue. Vital parameters were normal; liver enzymes and CRP were slightly elevated. Abdominal ultrasound demonstrated multiple hypoechogenic nodular liver lesions (Figure 1), while a chest radiograph revealed diffusely spread lung nodules. Subsequent investigations included an abdominal magnetic resonance imaging (MRI) (Figure 2), which showed multiple coalescent liver lesions with a target appearance, peripheral enhancement, and central necrosis. Whole-body positron emission tomography (PET)-contrast-enhanced (CE)-computed tomography (CT) again showed diffusely spread lung nodules and multiple osteolytic bone lesions (Figure 3). US guided liver biopsies initially showed aberrant microcirculation, and the possibility of a sampling error was suggested. Subsequently, a laparoscopic partial resection of one lesion was performed. The resected tissue revealed the same histopathological pattern, and the diagnosis of hepatic epithelioid hemangioendothelioma (HEHE) was made.

## Comment

HEHE is a rare, malignant, hepatic vascular tumor. A higher incidence in female patients (3:2) has been reported with a peak incidence during the fourth and fifth decades. The clinical presentation of the disease is highly variable with some patients being asymptomatic, while others experience fatigue, weight loss, and/or dull right upper quadrant pain. Liver tests may sometimes be elevated. Since many patients are asymptomatic or present with aspecific symptoms, lesions are often detected incidentally.

HEHE shares many imaging features with metastatic liver disease, which is much more frequent and is therefore often misdiagnosed.

On MRI of the liver, HEHE mostly presents as multifocal, subcapsular, confluent lesions with a target-like pattern of enhancement. Larger, subcapsular lesions often cause capsular retraction. The final diagnosis should be made on a pathological exam, but even then, diagnosis can be challenging. Therefore, suggesting the differential diagnosis of HEHE in the radiology report may help the pathologist and avoid the need for more extensive laparoscopic biopsies. Management of (H)EHE requires a multidisciplinary approach, preferably in a referral center [1].
